# DeepSUVR: Using temporal constraints to improve SUVR and Centiloid quantification

**DOI:** 10.1002/alz.091094

**Published:** 2025-01-09

**Authors:** Pierrick Bourgeat, Jurgen Fripp, Ashley G Gillman, Timothy Cox, Manu S. Goyal, Duygu Tosun, Pamela LaMontagne, Tammie L.S. Benzinger, Michael S. W. Weiner, Victor L Villemagne, Colin L Masters, Christopher C. Rowe, Vincent Dore, for the Alzheimer’s Disease Neuroimaging Initiative, OASIS3, and the AIBL research group

**Affiliations:** ^1^ CSIRO, Brisbane, QLD Australia; ^2^ CSIRO Health and Biosecurity, Australian E‐Health Research Centre, Brisbane, QLD Australia; ^3^ CSIRO Health and Biosecurity, Australian E‐Health Research Centre, Townsville, QLD Australia; ^4^ The Australian e‐Health Research Centre, Commonwealth Scientific and Industrial Research Organisation, Melbourne, VIC Australia; ^5^ Washington University School of Medicine in St. Louis, St. Louis, MO USA; ^6^ Department of Veterans Affairs Medical Center, Northern California Institute for Research and Education (NCIRE), San Francisco, CA USA; ^7^ Washington University School of Medicine, Saint Louis, MO USA; ^8^ Washington University in St. Louis, St. Louis, MO USA; ^9^ University of California, San Francisco, San Francisco, CA USA; ^10^ University of Pittsburgh, Pittsburgh, PA USA; ^11^ Florey Institute of Neuroscience and Mental Health, Parkville, VIC Australia; ^12^ Austin Health, Heidelberg, VIC Australia; ^13^ CSIRO Health and Biosecurity, Australian E‐Health Research Centre, Parkville, VIC Australia; ^14^ Department of Molecular Imaging, Austin Health, Melbourne, VIC Australia

## Abstract

**Background:**

PET quantification using the Standardised Uptake Value Ratio (SUVR) relies on the availability of a robust reference region. Intrinsic noise, spill in, and specific binding in the reference region can impact the reliability of the resulting SUVR. We evaluate a novel deep learning method trained on longitudinal data that penalises unexpected temporal changes and learns a SUVR correction factor that compensates for any noise or bias in the reference region, resulting in an improved quantification.

**Method:**

3098 participants with 2+ visits in AIBL/ADNI/OASIS (8826 scans) had their Amyloid PET images spatially normalised and quantified using the Centiloid SPM pipeline. A deep learning network (DeepSUVR) was trained to predict a SUVR correction factor (CF) for each spatially normalised image. For each iteration, the prediction was run on 2 random timepoints from the same participant, resulting in 2 Adjusted Centiloids computed using SUVR*CF and each tracer’s standard Centiloid transform. A loss function was defined to penalise unexpected temporal changes: Centiloid decreasing over time, Centiloid deviating from the curve defining the change in Centiloid/Year given the mean Centiloid. The loss also included a penalty for over‐correction (Slope of the linear regression between original and adjusted Centiloid should be 1). The model was trained using a subset of ADNI/AIBL (2037 participants) and evaluated in terms of longitudinal consistency on the remaining ADNI/AIBL participants and on OASIS data. The correlations between head‐to‐head tracers in the OASIS and GAAIN calibration datasets were also computed.

**Result:**

DeepSUVR increased the correlation between each pair of 11C‐PIB/18F‐Tracers in the GAAIN calibration dataset (Figure 1) and between pairs of 11C‐PIB/18F‐Florbetapir in OASIS (from R^2^=0.86 to R^2^=0.92) (Figure 2). Using DeepSUVR, the correlation between Centiloid/Year vs mean Centiloid increased from ρ=0.39 to ρ=0.48 in AIBL/ADNI and ρ=0.47 to ρ=0.51 in OASIS (Figure 3).

**Conclusion:**

We have proposed a novel deep learning technique to correct SUVR estimation based on noise in longitudinal trends. The longitudinal constraint led to higher agreement between tracers in the head‐to‐head GAAIN/OASIS datasets and higher longitudinal consistency. Importantly, while the model was trained using pairs of images, the prediction is run on single images. Future work will evaluate this approach on tau tracers.

